# First insights on the retroelement *Rex1* in the cytogenetics of frogs

**DOI:** 10.1186/s13039-015-0189-5

**Published:** 2015-11-05

**Authors:** Juliana Nascimento, Diego Baldo, Luciana Bolsoni Lourenço

**Affiliations:** Departamento de Biologia Estrutural e Funcional, Instituto de Biologia, Universidade Estadual de Campinas, 13083-863 Campinas São Paulo, Brazil; Laboratorio de Genética Evolutiva, Instituto de Biología Subtropical (CONICET-UNaM), Facultad de Ciencias Exactas Químicas y Naturales, Universidad Nacional de Misiones, Félix de Azara 1552, CPA N3300LQF Posadas, Misiones Argentina

**Keywords:** Repetitive DNA, in situ hybridization, Chromosome, Retrotransposon, Leptodactylidae

## Abstract

**Background:**

While some transposable elements (TEs) have been found in the sequenced genomes of frog species, detailed studies of these elements have been lacking. In this work, we investigated the occurrence of the *Rex1* element, which is widespread in fish, in anurans of the genus *Physalaemus*. We isolated and characterized the reverse transcriptase (RT)-coding sequences of *Rex1* elements of five species of this genus.

**Results:**

The amino acid sequences deduced from the nucleotide sequences of the isolated fragments allowed us to unambiguously identify regions corresponding to domains 3–7 of RT. Some of the nucleotide sequences isolated from *Physlaemus ephippifer* and *P. albonotatus* had internal deletions, suggesting that these fragments are likely not active TEs, despite being derived from a *Rex1* element. When hybridized with metaphase chromosomes, *Rex1* probes were revealed at the pericentromeric heterochromatic region of the short arm of chromosome 3 of the *P. ephippifer* karyotype. Neither other heterochromatin sites of the *P. ephippifer* karyotype nor any chromosomal regions of the karyotypes of *P. albonotatus, P. spiniger* and *P. albifrons* were detected with these probes*.*

**Conclusions:**

*Rex1* elements were found in the genomes of five species of *Physalaemus* but clustered in only the *P. ephippifer* karyotype, in contrast to observations in some species of fish, where large chromosomal sites with *Rex1* elements are typically present.

**Electronic supplementary material:**

The online version of this article (doi:10.1186/s13039-015-0189-5) contains supplementary material, which is available to authorized users.

## Background

Eukaryotic genomes contain large amounts of repetitive DNA sequences, many of which are interspersed repeats derived from transposable elements (TEs) (reviewed in [1–3]). With the ability to integrate and occupy a large portion of the eukaryotic genome, TEs greatly influence genomic architecture (reviewed in [1, 4]). TEs are also involved in karyotype evolution because these mobile sequences can induce chromosomal rearrangements, including deletions, duplications, inversions and translocations (reviewed in [1, 5]). Therefore, the identification of this type of repetitive sequence may be valuable for evolutionary cytogenetic studies.

TE sequences are grouped in two large families, class I elements (retrotransposons) and class II elements (transposons), which are characterized by the intermediate molecule used in the transposition process. Class I elements have an RNA molecule as an intermediary for transposition, while class II elements move in the genome using DNA copies as intermediates or without any intermediate [2, 6, 7]. The eukaryotic transposons are further classified as “cut-and-paste” transposons, *Helitrons* and *Politrons*, which are, respectively, non-replicative, rolling-circle replicative and self-synthesizing (reviewed in [2, 3]). Among the eukaryotic retrotransposon, two principal groups are recognized, according to the presence or absence of long terminal repeats (LTR) flanking their open ready frames (ORFs): the LTR retrotransposons and the non-LTR retrotransposons, also known as LINEs (long interspersed nucleotide element). Additionally, *Penelope* and *DIRS* (*Dictyostelium* intermediate repeat sequence) retrotransposons have been identified (reviewed in [2]).

The retrotransposons families *Rex* (Retroelement of *Xiphophorus*) *1*, *Rex2*, *Rex3* and *Rex6* are non-LTR retrotransposons, and they were first isolated from the fish genus *Xiphophorus* [8–10]. The *Rex1* element encodes a reverse transcriptase and an apurinic/apyrimidinic endonuclease, which is frequently lost [9]. The 3′ untranslated region of several *Rex1* elements is followed by tandem repeat oligonucleotides that are variable in length (5–7 nt) and sequence. Based on an analysis of RT amino acid sequence, Volff and colleagues [9] reasoned that the *Rex1* sequences and *Babar* elements (for *Battrachocottus baikalensis* retrotransposon) cannot be assigned to any other known family of TE and suggested a moderately close relationship between *Rex1/Babar* elements and members of the *CR1* family of non-LTR retrotransposons.

The *Rex* family is widespread in fishes and was already mapped to a number of fish karyotypes through in situ hybridization, providing valuable markers for karyotype comparisons (examples in [8–20]; for review, see [21]). For Anura, the only available reports on *Rex* sequences arose from studies not designed specifically for the analysis of this TE but rather from the sequencing of the whole genomes of the pipid *Xenopus tropicalis* [22, 23] and the dicroglossid *Nanorana parkeri* [24; GenBank accession number: JYOU00000000.1], which are representatives of the non-neobatrachian and Ranoidea, respectively. In addition, in contrast to fish, the karyotype organization of this or any other TE has not been explored using cytogenetic studies.

In this study, we aimed to evaluate i) whether *Rex1* is also present in Hyloidea, the Anuran superfamily that, together with Ranoidea, composes Neobatrachia and ii) whether *Rex1* sequences are sufficiently clustered in Anuran genomes to be used as chromosomal markers in Anuran cytogenetics. To assess these goals, we elected the leptodactylid genus *Physalaemus*, which comprises 46 species [25] that are currently arranged in two major clades: the *P. cuvieri* Clade with six species groups (*P. biligonigerus* group, *P. cuvieri* group, *P. gracilis* group, *P. henselii* group, *P. olfersii* group) and the *P. signifer* Clade [26]. Twenty of the species of *Physalaemus* have already been karyotyped [27–37], and the results show 2n = 22. *Physalaemus* is attractive for cytogenetic and genomic studies, particularly because of the high interspecific variation in the number and/or distribution of nucleolus organizer regions (NOR) [29, 30, 32–37]) and because heteromorphic sex chromosomes are only recognized in *P. ephippifer* [34]. We searched for sequences related to the *Rex1* family in five species belonging to different species groups of *Physalaemus* and used the isolated sequences as probes for in situ hybridization assays.

## Results

### *Rex1* sequences of *Physalaemus* species

We obtained 23 clones containing fragments of *Rex1* isolated from genomic DNA of *Physalaemus ephippifer*, 18 from *P. albifrons*, 16 from *P. albonotatus* and one from *Physalaemus* aff. *cuvieri*. One sequence of *P. henselii* and one of *P. spiniger* were isolated and sequenced directly without cloning.

**Fig. 1:**
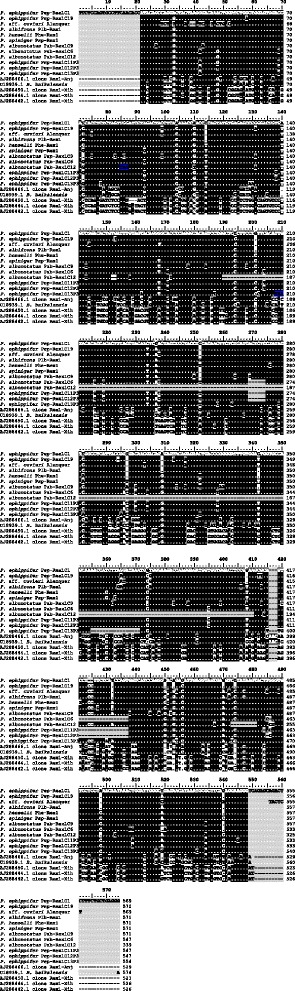
Alignment of the *Rex1* fragments isolated from species of *Physalaemus* with corresponding sequences available in GenBank. The sequences *P. ephippifer* Pep-Rex1C11P3, *P. ephippifer* Pep-Rex1C12P3 and *P. ephippifer* Pep-Rex1C13P3 were obtained from the microdissected 3p *per* band. The primers used to isolate the sequences are shaded in gray at the ends of the sequences. Black areas indicate identical sites, while variable sites are colored white. Premature stop codons are shown in blue. U18939.1 is the GenBank accession number of a *Babar* sequence (a *Rex1*-related element) of *Battrachocottus baikalensis*. AJ288466.1, AJ288450.1, AJ288444.1, and AJ288462.1 are GenBank accession numbers of retroelement *Rex1* sequences isolated from *Anguilla japonica* (AJ288466.1) or *Xiphophorus helleri*

Among the 16 fragments isolated from *Physalaemus albonotatus*, three types of sequences were found, which differed in nucleotide sequence and size. In the 339 bp sequence isolated from the *P. albonotatus* genome (Pab-Rex1C12), a 221 bp segment (positions 190–410 in Fig. 1) is missing, which was present in all of the remaining sequences isolated from the *Physalaemus* species. The first 190 bp and the last 160 bp of this sequence were highly similar to the corresponding regions of the other isolated sequences (Fig. 1). A codon analysis of this truncated sequence of *P. albonotatus* revealed an in-frame stop codon at the beginning of the isolated sequence (at positions 85–87 as shown in Fig. 1). The other two sequences isolated from *P. albonotatus* were 86 % similar to each other and were 547 bp and 571 bp in length. One segment with 6 nucleotides and another with 17 nucleotides that were present in the 571 bp fragments were absent in the 547 bp sequence (segments from positions 269–274 and from positions 421–437 as shown in Fig. 1). Two sequences that were 95 % similar to each other were also recognized among the fragments isolated from *P. ephippifer* (Fig. 1).

The *Rex1* fragments isolated from all the *Physalaemus* species, except for the 339 bp sequence of *P. albonotatus*, were very similar to each other (average similarity = 92 %) and were 68 and 73 % similar to the *Rex1/Babar* sequences already described for *Anguilla japonica and Battrachocottus baikalensis*, respectively (Fig. 1). We note that the sequence isolated from *Physalaemus* aff. *cuvieri* differed significantly at positions 29–52 with regard to the other sequences of *Physalaemus* (Fig. 1). When the *Rex1* sequences of *Physalaemus* (except for the 339 bp sequence of *P. albonotatus*) were compared with the sequence *REX1-5_XT*, which was obtained from the anuran *Xenopus tropicalis* and previously recognized as *Rex1* [22, 23] (sequence available at Repbase database http://www.girinst.org/censor/index.php), lower similarity values were found (from 54 to 58 %) (Additional file 1). Comparison of the *Rex1* sequences isolated from *Physalaemus* with the element *CR1* of *Gallus gallus*, which Volff and colleagues consider to be distantly related to *Rex1/Babar* elements [9], showed no similarity (Additional file 1).

**Fig. 2:**
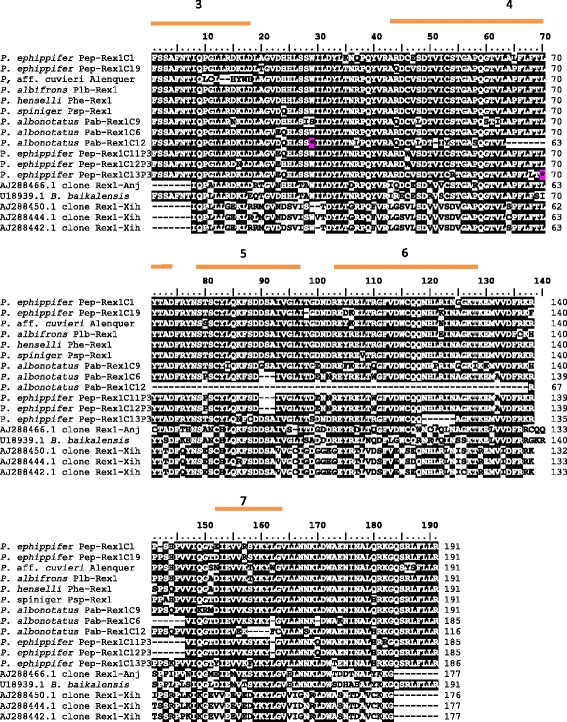
Amino acid sequence inferred from the nucleotide sequences shown in Fig. 1. Black sites are conserved amino acids. Bars indicate domains 3–7 of the *Rex1* RT as determined by Volff and colleagues [8, 9] and Malik and colleagues [37]. Asterisks represent premature stop codons

Amino acid sequence translation of all fragments of *Rex1* (Fig. 2) allowed us to clearly identify the regions corresponding to the conserved domains 3 to 7 of the RT, as identified by Malik and colleagues [37] and Volff and colleagues [8, 9]. When compared to *Battrachocottus baikalensis* (GenBank accession number U18939.1) and *Anguilla japonica* (GenBank accession numbers AJ288466.1) sequences, the *Rex1* sequences isolated from *Physalaemus ephippifer*, *P. albifrons*, *P. henselli*, *P. spiniger*, *P. albonotatus* and *P*. aff. *cuvieri* from Alenquer–PA failed to show an AAC nucleotide triplet at positions 416–418 of the sequences shown in Fig. 1, as well as of the sequences isolated from *Xiphophorus helleri* [9] (GenBank accession numbers AJ288450.1, AJ288444.1 and AJ288442.1). The absence of this ACC triplet potentially affects the translation of two codons. In addition, deletions of one or two nucleotides could be detected in the fragments Pep-Rex1C1, Pep-Rex1C17 and Pep- Rex1C19 of *P. ephippifer* after comparing these sequences to the others (Fig. 1).

### Mapping of *Rex1* sequences on metaphase chromosomes

**Fig. 3:**
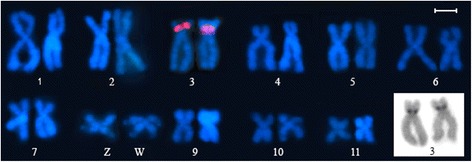
Chromosome localization of the Pep-RexC17 sequence in the *Physalaemus ephippifer* karyotype. Note the hybridization signal in chromosome pair 3. The FITC-signals are shown in red for better visualization. The C-banded pair in the inset shows the band that coincides with the site detected by the *Rex1* probe and was thus microdissected for the isolation of some of the *Rex1* sequences shown in Fig. 1. Bar = 1 μm

When used as probes and mapped to metaphase chromosomes, the *Rex1* sequences did not reveal any chromosomal sites at all (not even small dots) in the karyotypes of *Physalaemus albonotatus, P. albifrons, P.* aff. *cuvieri* or *P. spiniger.* In contrast, the probe Pep-Rex1C17 localized to the pericentromeric region of the short arm of chromosome pair 3 (3p *per*) of *P. ephippifer* (Fig. 3), suggesting accumulation of *Rex1* sequences at this heterochromatic chromosomal region (Fig. 3 – inset). No hybridization signal was observed at any other site of this karyotype (Fig. 3), not even at regions previously revealed to be heterochromatic by Nascimento and colleagues [34]. The region at 3p *per* also hybridized with type I 5S ribosomal DNA (5S rDNA), as previously reported by Nascimento and colleagues [34].

### *Rex1* and 5S rDNA sequences isolated from microdissected 3p *per* of *P. ephippifer*

Polymerase chain reaction (PCR) using primers for *Rex1* sequences resulted in the isolation of ~550-bp fragments from the microdissected 3p *per* band of the *Physalaemus ephippifer* karyotype, and three of these fragments were cloned and sequenced. Two cloned fragments were 547 bp (*P. ephippifer* Pep-Rex1C11P3 and *P. ephippifer* Pep-Rex1C12P3 in Fig. 1), and one was 554 bp (*P. ephippifer* Pep-Rex1C13P3 in Fig. 1). All of these fragments were very similar to the major *Rex1* sequences isolated from genomic DNA of *Physalaemus* species (Fig. 1). The 547-bp fragments and the sequence Pab-Rex1C6 of *P. albonotatus* had a deletion of two segments compared to the other sequences (from position 269 to position 274, and from position 421 to position 437 in Fig. 1).

**Fig. 4:**
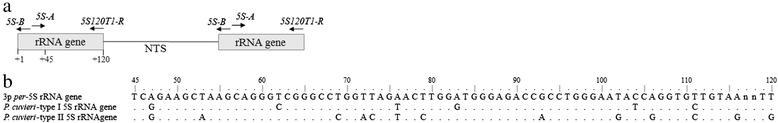
Analysis of the 5S rDNA of the 3p *per* band of the *Physalaemus ephippifer* karyotype. **a** Annealing sites of the primers used for the analysis of the 5S rDNA (for details, see Methods section). NTS: non-transcribed spacer. **b** Alignment of the nucleotide sequence of a fragment of the 5S rRNA gene isolated from the 3p *per* band of the *Physalaemus ephippifer* karyotype with the type I and type II 5S rRNA gene of *Physalaemus cuvieri* (GenBank accession numbers: JF281131 and JF281133, respectively). The site positions were numbered according to the scheme shown in **a**

PCR using primers for 5S rDNA resulted in the amplification of ~300-bp fragments from the microdissected 3p *per* band. Their nucleotide sequencing proved the presence of the 5S rRNA gene in this chromosome band (Fig. 4).

## Discussion

The results described here suggest that the elements we isolated from the genus *Physalaemus* might represent part of the retroelement *Rex1*, or at least sequences derived from it. We have formed this hypothesize because the elements’ nucleotide sequences and presumed amino acid sequences are highly similar to the previously described RT coding region sequences of *Rex1*, especially those from *Anguilla japonica* [9] and *Battrachocottus baikalensis* (GenBank accession number U18939.1 – see comment in Volff and colleagues [9]). Although Volff and colleagues [9] affirm that *Rex1/Babar* elements are somewhat related to the *CR1* element isolated from *Gallus gallus*, we could not detect any similarity between this element and the sequences isolated from species of *Physalaemus*.

The nucleotide sequences isolated from chromosome 3 of *Physalaemus ephippifer* and two of the three fragments isolated from *P. albonotatus* were similar to the RT-coding sequences of the *Rex1* elements; however, these sequences have deletions that may affect their presumed reading frame. For the sequence Pab-Rex1C12 of *P. albonotatus*, a premature stop codon was also detected. It is likely, therefore, that these sequences are no longer active retroelements. Interestingly, the truncated sequences isolated from *P. ephippifer* were isolated from the heterochromatic block at 3p per, and this chromosomal region was detected using *Rex1* probes in fluorescence in situ hybridization (FISH) assays. It is therefore reasonable to suggest that the loss of transposition ability may be related to mechanisms of accumulation of *Rex1*-derived sequences in this heterochromatic region. The accumulation of TEs in heterochromatin has been reported and discussed by some authors [4, 19, 38–41]. Charlesworth & Langley [38] report that the suppression of heterochromatin crossing-over and gene inactivity are two factors that influence the preferential distribution of transposable sequences in such regions of the genome. For *P. ephippifer*, the molecular mechanisms involved in *Rex1*-derived sequences in 3p *per* were not investigated, but co-localization with 5S rDNA in FISH assays was an intriguing finding.

The association between TEs and rDNA sequences was already reported for many organisms. In arthropods, for example, R1 and R2 retrotransposable elements are components of the major rDNA [42, 43]. In the fish *Erythrinus erythrinus*, sequences belonging to the *Rex3* family were mapped at 5S rDNA sites and proposed to be involved in the spreading of 5S rDNA sequences in the genomes of some karyomorphs [43]. No evidence that supports the involvement of *Rex1* sequences in spreading of 5S rDNA in *Physalaemus* or any other anuran is available to date. The molecular nature of the association between these sequences (whether they are interspersed or form independent clusters that are sufficiently close to co-locate in FISH assays) as well as the evolutionary implications of this association are open questions for future studies.

Although in situ hybridization revealed a cluster of *Rex1* sequences at the heterochromatic band in the short arm of chromosome 3 of *Physalaemus ephippifer*, no other heterochromatic site in the karyotype of this species was detected by *Rex1* probes in FISH experiments. *Rex1* probes were unable to detect any chromosomal site in the karyotypes of *P. albonotatus, P. spiniger*, or *P. albifrons.* These findings diverge from those found for several species of fish, in which *Rex1*, *Rex3* and *Rex6* elements have been mapped to several heterochromatin sites [13, 14, 16, 19]. The FISH experiments suggest that in *Physalaemus* species, *Rex1* sequences are less abundant than those found for fish species. It is noteworthy that in the genome of anuran *Xenopus (Silurana) tropicalis*, non-LTR retrotransposons, including *Rex1*, *CR1* and *L2* elements, are estimated to correspond to only 0.6 % of the genome [23].

## Conclusions

Our findings show that the *Rex1* family of retrotransposons is not restricted to *Xenopus tropicalis* and *Nanorana parkeri* but is also present in the leptodactylid genus *Physalaemus*. The occurrence of these TEs both in two basal lineages of Anura (*i.e.*, pipids and dicroglossids) and in a derived genus of Neobatrachia (*i.e.*, *Physalaemus*) suggests that this element could be largely distributed in anuran genomes. Although *Rex1* sequences were not highly clustered in the *Physalaemus* karyotypes, which differed from observations in fish species, we provided evidence for the accumulation of *Rex1* sequences in a heterochromatin site of the karyotype of *P. ephippifer* that associated with 5S rDNA sequences. Our results shed new light for further investigation into the evolutionary dynamics of both types of sequences in anuran genomes.

## Methods

### Samples

Individuals were used that belonged to the two major clades of *Physalaemus* (*i.e.*, *P. cuvieri* Clade and *P. signifer* Clade [26]). Samples of liver and muscle of *P. albonotatus* (*P. cuvieri* group), *P. albifrons* (*P. cuvieri* group), *P.* aff. *cuvieri* (*P. cuvieri* group), *P. ephippifer* (*P. cuvieri* group), *P. spiniger* (*P. signifer* Clade) and *P. henselii* (*P. henselii* group of the *P. cuvieri* Clade) were obtained from the tissue collection deposited at the Laboratory of Chromosomal Studies, at the Department of Structural and Functional Biology of the Institute of Biology, at the University of Campinas (UNICAMP) (Table 1). Metaphase chromosome preparations of *P. ephippifer* individuals from Belém-PA (ZUEC 13734♂; ZUEC 13740♂, ZUEC13741♀ and ZUEC 13705♂) were obtained from cell suspensions available at the same laboratory, previously obtained by Nascimento and colleagues [34]. The experiments were approved by the ethics committee CIBio/IB-UNICAMP (#2005/03).

**Table 1:** Identification, voucher number and locality of the specimens used for the isolation of the *Rex1* fragments

*Species*	*Specimen voucher*	Locality
*Physalaemus albifrons* (*P. cuvieri* group)	ZUEC 12361	Vassouras, Barreirinhas-MA, Brazil
*Physalaemus albonotatus* (*P. cuvieri* group)	ZUEC 16419	Lambari do Oeste-MT, Brazil
*Physalaemus ephippifer* (*P. cuvieri* group)	ZUEC 13705	Belém-PA, Brazil
*Physalaemus* aff. *cuvieri* (*P. cuvieri* group)	ZUEC 18191	Alenquer-PA, Brazil
*Physalaemus henselii* (*P. henselii* group)	MHNM 9512	Ruta 5, km 492, Pueblo Madera, Rivera, Uruguay
*Physalaemus spiniger* (*P. signifer* Clade)	ZUEC 14516	Reserva Salto Morato-Curitiba-PR, Brazil

*ZUEC*: Museu de Zoologia “Prof. Adão José Cardoso”, Universidade Estadual de Campinas (UNICAMP), Campinas, São Paulo, Brasil. *MNHN*: Museo Nacional de Historia Natural de Montevideo. Uruguay. PA: State of Pará; *MT*: State of Mato Grosso; *MA*: State of Maranhão; *PR*: State of Paraná

### Isolation of partial sequence of the retrotransposon Rex1 from genomic DNA

Samples of genomic DNA were isolated according to a procedure previously reported by Medeiros and colleagues [44] and subjected to PCR to isolate *Rex1* sequences. PCR assays were performed using the primers *RTX1-F1* (TTCTCCAGTGCCTTCAACACC) and *RTX1-R3* (TCCCTCAGCAGAAAGAGTCTGCTC) [9], which are specific for isolating a region of the gene encoding the RT (ORF 2) of the TE *Rex1*. The products of these reactions were analyzed after electrophoresis in a 1 % agarose gel. Bands corresponding to fragments of approximately 550 bp were observed in each case. For *Physalaemus albonotatus*, an additional band of fragments of approximately 350 bp was obtained. All of these bands were cut from the gel with sterile blades, and the DNA fragments were purified with the GFX PCR and Gel Band DNA Purification kit (GE Healthcare).

### Cloning of fragments of the retrotransposon Rex1 isolated from genomic DNA

The *Rex1* fragments amplified as described above were each inserted into the plasmid vector pGEM-T (Promega), following the manufacturer’s instructions. The recombinant vectors were used to transform JM109 *Escherichia coli* competent cells using the cloning kit Transformaid Bacterial Transformation (Fermentas), following the manufacturer’s directions. The positive clones were selected and used for extraction of plasmids, according to the mini-prep method described by Sambrook and colleagues [45]. For amplification of the inserts, samples of the isolated plasmids were used in PCR with T7 and SP6 universal primers. After purification with the GFX PCR and Gel Band DNA Purification kit (GE Healthcare), samples of the amplified inserts were sequenced using the BigDye Terminator kit (Applied Biosystems), according to the manufacturer’s instructions. The sequences obtained were edited using BioEdit software [46], and compared to sequences available at the GenBank and Repbase databases. For comparison to sequences in the Repbase database, we used the CENSOR engine with default parameters, searching within the *Xenopus (Silurana) tropicalis* collection for *Rex1*.

### Metaphase chromosome analyses

To obtain metaphase chromosome preparations of *Physalaemus ephippifer* (ZUEC 13740 and ZUEC 13741), cell suspensions available at the Laboratory of Chromosomal Studies, at the Department of Structural and Functional Biology of the Institute of Biology, at the University of Campinas (UNICAMP) were dropped onto clean slides. Cloned *Rex1* fragments generated from genomic DNA of *Physalaemus* species were labeled with dUTP-biotin (Roche®) by PCR and *in situ* hybridized to the karyotypes. The hybridization protocol [47] used an anti-biotin antibody (Vector) and a fluorescein isothiocyanate (FITC)-conjugated secondary antibody (Vector). The chromosomes were stained with DAPI (0.5 μg/mL).

### Isolation of *Rex1* and 5S rDNA sequences from the microdissected 3p *per* band of *Physalaemus ephippifer*

To demonstrate that *Rex1* sequences are present in the pericentromeric C-band of the short arm of chromosome 3 of *Physalaemus ephippifer* (as suggested by FISH - see Results for details), which also bears 5S rDNA (as reported previously [34]), we isolated *Rex1* and 5S rDNA sequences from the microdissected 3p *per* band by PCR. For microdissection, cell suspensions of the *P. ephippifer* specimen ZUEC 13734 were dropped onto a membrane strip containing polyethylene-naphthalate (PEN) that was previously exposed to UV and incubated at −20 °C for 30 min. The material was subjected to C-banding according to a previously reported procedure [48]. The best metaphase examples were used for UV laser-microdissection of 18 copies of the heterochromatic pericentromeric region of the short arm of chromosome 3 of *P. ephippifer* with the MicroBeam 4.1 system (Zeiss). The cuts in the PEN membrane were made with UV at 0.5–0.6 μJ/pulse, and isolated regions were catapulted using a pulse of 0.2 μJ to the lid of a microtube containing 9 μL of TE buffer. The collected material was maintained in TE for at least 16 h and then subjected to PCR using the primers *RTX1-F1* and *RTX1-R3* [9] for the amplification of *Rex*1 sequences and the primers *5S-A* (TACGCCCGATCTCGTCCGATC) and *5S-B* (5′-CAGGCTGGTATGGCCGTAAGC-3′) [49] for the amplification of 5S rDNA sequences. The amplified *Rex1* fragments were cloned and sequenced as described above. Two rounds of PCR with the primers *5S-A* and *5S-B* were performed, and the amplified 5S rDNA fragments were directly sequenced using the primers *5S-A* [49] and *5S120T1-R* (AGCTTACAGCACCTGGTATTC) [50] (see the annealing sites of the primers in Fig. 4) and the BigDye Terminator kit (Applied Biosystems), according to the manufacturer’s instructions.

**Fig. 5:**
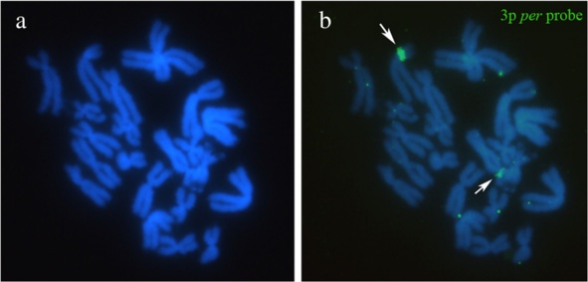
*In situ* hybridization of a probe generated from the microdissection of the 3p *per* band of the karyotype of *Physalaemus ephippifer*. **a** DAPI image. **b** Merged DAPI and FISH images. Only the 3p *per* band (*arrows*) was detected

To ensure that the microdissected regions correspond to the 3p *per* band, we used the captured DNA as probes in FISH assays. Therefore, the microdissected material was first amplified using GenomePlex Single Cell WGA4 (Sigma-Aldrich) and labeled with dUTP-biotin (Roche®) using GenomePlex Single Cell WGA3 (Sigma-Aldrich) (Fig. 5). The hybridization and probe detection protocols were the same as used with the *Rex1* probes (reported above).

## Additional file

Additional file 1:
**Alignment of the fragments of the retroelement** ***Rex1*** **isolated from species of** ***Physalaemus*** **with corresponding sequences available in GenBank and Repbase.** Note that the sequence CR1 of *Gallus gallus* (GenBank accession number U88211.1) significantly differs from all other sequences. The primers used to isolate the sequences are indicated in gray. Black areas indicate identical sites, while variable sites are colored white. Premature stop codons are shown in blue. U18939.1 is the GenBank accession number of a *Babar* sequence (a *Rex1*-related element) of *Battrachocottus baikalensis*. AJ288466.1, AJ288450.1, AJ288444.1 and AJ288442.1 are GenBank accession numbers of retroelement *Rex1* sequences isolated from *Anguilla japonica* (AJ288466.1) or *Xiphophorus helleri*. *Xenopus tropicalis* REX1-5, REX1-2 and REX1-3 are sequences isolated from *Xenopus tropicalis* and available at the Repbase database (http://www.girinst.org/censor/index.php). (DOCX 33 kb)
