# Supplementary material for: First insights on the retroelement Rex1 in the cytogenetics of frogs
Source: Mol Cytogenet. 2015 Nov 5;8:86. doi: 10.1186/s13039-015-0189-5 (PMC4635592; doi:10.1186/s13039-015-0189-5)
Supplement: Additional file 1: — Alignment of the fragments of the retroelement Rex1 isolated from species of Physalaemus with corresponding sequences available in GenBank and Repbase. Note that the sequence CR1 of Gallus gallus (GenBank accession number U88211.1) significantly differs from all other sequences. The primers used to isolate the sequences are indicated in gray. Black areas indicate identical sites, while variable sites are colored white. Premature stop codons are shown in blue. U18939.1 is the GenBank accession number of a Babar sequence (a Rex1-related element) of Battrachocottus baikalensis. AJ288466.1, AJ288450.1, AJ288444.1 and AJ288442.1 are GenBank accession numbers of retroelement Rex1 sequences isolated from Anguilla japonica (AJ288466.1) or Xiphophorus helleri. Xenopus tropicalis REX1-5, REX1-2 and REX1-3 are sequences isolated from Xenopus tropicalis and available at the Repbase database (http://www.girinst.org/censor/index.php). (DOCX 33 kb) [file 13039_2015_189_MOESM1_ESM.docx]

**10 20 30 40 50 60 70**

**....|....|....|....|....|....|....|....|....|....|....|....|....|....|**

*P. ephippifer* Pep-Rex1C1 **TTCTCCAGTGCCTTCAACACCATACAGCCAGGACTACTAAGGGACAAACTGGATCTGGCTGGAGTGGACC** 70

*P. ephippifer* Pep-Rex1C19 **TTCTCCAGTGCCTTCAACACCATACAGCCAGGGCTACTAAGGGACAAACTGGATCTGACTGGAGTGGACC** 70

*P.* aff. *cuvieri* Alenquer **TTCTCCAGTGCCTTCAACACCATACAGCTCTGTCTAAT--GTCACTACTGGCATCTGGCTGGAGTGGACC** 68

*P. albifrons* Plb-Rex1 **TTCTCCAGTGCCTTCAACACCATACAGCCAGGGCTACTAAGGGACAAACTGGATCTGGCTGGAGTGGACC** 70

*P. henselli* Phe-Rex1 **TTCTCCAGTGCCTTCAACACCATACAGCCAGGGCTACTAAGGGACAAACTGGATCTGGCTGGAGTGGACC** 70

*P. spiniger* Psp-Rex1 **TTCTCCAGTGCCTTCAACACCATACAGCCAGGGCTACTAAGGGACAAACTGGATCTGGCTGGAGTGGACC** 70

*P. albonotatus* Pab-Rex1C9 **TTCTCCAGTGCCTTCAACACCATACAACCAGGGCTACTAAGGAACAAACTGGATCTGGCTGGAGTGGACC** 70

P. albonotatus Pab-Rex1C6 **TTCTCCAGTGCCTTCAACACCATACAGCCAGGGCTACTAAGAGACAAGCTGGATCTGGCTGGAGTGGAGC** 70

*P. albonotatus* Pab-Rex1C12 **TTCTCCAGTGCCTTCAACACCATACAGCCAGGGCTACTAAGGGACAAGCTGGATCTGGCTGGAGTGGACC** 70

*P. ephippifer* Pep-Rex1C11P3**TTCTCCAGTGCCTTCAACACCATACAGCCAGGGCTACTAAGAGACAAGCTGGATCTGGCTGGAGTGGAGC** 70

*P. ephippifer* Pep-Rex1C12P3**TTCTCCAGTGCCTTCAACACCATACAGCCAGGGCTACTAAGAGACAATCTGGATCTGGCTGGAGTGGAGC** 70

*P. ephippifer* Pep-Rex1C13P3**TTCTCCAGTGCCTTCAACACCATACAGCCAGGGCTACTAAGAGACAAACTGGATCTGGCTGGAGTGGAGC** 70

AJ288466.1 clone Rex1-Anj  **---------------------ATACAGCCTGCGCTTCTGAGGGACAAGTTGGACCGCACAGGGGTGAACC** 49

U18939.1 *B. baikalensis* **TTCTCCAGTGCCTTCAACACCATTCAGCCTTTGCTTCTGAGGGACAAGCTGGAGCAGACCGGGGTGGACC** 70

AJ288450.1 clone Rex1-Xih **---------------------ATCCAGCCTCTGCTACTGGGTGAGAAGCTGCGGAGGATGGGTGTCAACG** 49

AJ288444.1 clone Rex1-Xih **---------------------ATCCAGCCTCTGCTGCTGGGTGAGAAGCTGCGGTTGATGGGTGTCAACG** 49

AJ288442.1 clone Rex1-Xih **---------------------ATCCAGCCTCTGCTACTGGGTGAGAAGCTGCGGAGGATGGGTGTCGACG** 49

*Poeciliopsis gracilis* Rex1 **---------------------ATCCAGCCAGGATTACTGTGGGAGAAGCTGGCTAAAATGGACATAGATG** 49

*Xenopus* *tropicalis* REX1-5 **TTCTCCAGTGCCTTTAATACCATTCAACCGGCAGTATTGGGGGAGAAATTAAGGAACATAAACATTGATA** 70

*Xenopus* *tropicalis* REX1-2 **TACAGCTCTGCTTTCAACACCATCGTACCATCCAGACTTGTCATGAAACTCCGTGACCTGAACATCGGTT** 70

*Xenopus* *tropicalis* REX1-9 **TATAGCTCTGCATTCAATACCATCGTTCCCTCGAAACTGGACAGGAAACTACAAGATCTAGGATTGAGCA** 70

*Gallus gallus* CR1 **TCCACCAACGCCGTCACTCCATCATAGAAGGCCACAAGATTGGTTAAGCATGACCTTCCCCTGGTGAAGC** 70

**80 90 100 110 120 130 140**

**....|....|....|....|....|....|....|....|....|....|....|....|....|....|**

*P. ephippifer* Pep-Rex1C1 **ATCACCTCTCTAGTTGGATCCTAGACTACCTCAAAAACCAACCCCAGTATGTGAGAGCCCGGGACTGTGA** 140

*P. ephippifer* Pep-Rex1C19 **ACCACCTCTCTAGTTGGATCCTAGACTATCTCACAAACCGACCCCAGTATGTGAGAGCCCAGGACTGTGT** 140

*P.* aff. *cuvieri* Alenquer **ACCATCTCTCTAGTTGGATCCTAGACTATCTCACAAACCGACCCCAGTATGTGAGAGCCCGGGACTGTGT** 138

*P. albifrons* Plb-Rex1 **ACCACCTCTCTAGTTGGATCCTAGACTACCTCACAAACCGACCCCAGTATGTGAGAGCCCGGGACTGTGT** 140

*P. henselli* Phe-Rex1 **ACCACCTCTCTAGTTGGATCCTAGACTACCTCACAAACCGACCTCAGTATGTGAGAGCCCGGGACTGTGT** 140

*P. spiniger* Psp-Rex1 **AGCACCTCTCTAGTTGGATCCTGGACTACCTCACAAACCGACCTCAGTATGTGAGAGCCCGGGACTGTGT** 140

*P. albonotatus* Pab-Rex1C9 **ATCATCTCTCTATTTCGATCCTAGACTATCTCACAAACCGACCTCAGTATGTGAGAGCCCAGGACTGTGT** 140

*P. albonotatus* Pab-Rex1C6 **AGCACCTCTCTAGTTGGATCCTGGACTACCTCACAAACCGACCTCAGTATGTGAGAGCCCGGGACTGTGT** 140

*P. albonotatus* Pab-Rex1C12 **AGCACCTCTCTAGTTAGATCCTAGACTACCTCACAAACCTACCTCAGTATGTGAGAGCCCAGGACTGTGT** 140

*P. ephippifer* Pep-Rex1C11P3**AGCACCTCTCTAGTTGGATCCTGGACTACCTCACAAACCGACCTCAGTATGTGAGAGCCCGGGACTGGGT** 140

*P. ephippifer* Pep-Rex1C12P3**ATCACCTCTCTAGTTGGATCCTGGACTACCTCACAAACCGACCTCAGTATGTGAGAGCCCGGGACTGGGT** 140

*P. ephippifer* Pep-Rex1C13P3**AGCACCTTTCTAGTTGGATCCTAGACTACCTCACAAACCGACCTCAGTATGTGAGAGCAAGGGACTGTGT** 140

AJ288466.1 clone Rex1-Anj **ACCACCTCACAGCATGGATCCTGGACTACCTCACCGACCGACCACAGTATGTGAGGATACAGGACTGTGA** 119

U18939.1 *B. baikalensis* **ACCACCTCACTGCATGGATCCTGGACTACCTCACCAACCGTCCACAGTATGTGCGGATACGGGAGTGTGA** 140

AJ288450.1 clone Rex1-Xih **ACTCAGTGATCTCCTGN---ACTGACTACTTGACAGGCAGGCCACAGTTTGTCCGTCTGGGCAGTGTCCT** 116

AJ288444.1 clone Rex1-Xih **ACTCAGTGATCTCCTGGGTTACTGACTACTTGACAGGCAGGCCACAGTTTGTCCGTCTGGGCAGTGTCCT** 119

AJ288442.1 clone Rex1-Xih **ACTCAGTGATCTCCTGGGTWACTGACTACTTGACAGGCAGGCCACAGTTTGTCCGTCTGGGCAGTGTCCT** 119

*Poeciliopsis gracilis* Rex1 **CATCACTCATCACCTGGATCATCGACTATTTGACACCCAGACCCCAGTATGTGAGATTACAGAGCTGCAA** 119

*Xenopus* *tropicalis* REX1-5 **TCCGCTTGGTCTCATGGATTATGGATTATTTGACCTGTCGTCCTCAATATGTCCGACTACAGAACTGTGT** 140

*Xenopus* *tropicalis* REX1-2 **CCTCCCTGTGCAGCTGGATCCTGGACTTCCTGACAAACAGACCTCAGGTGGTTCGGATCGGCAACATCAC** 140

*Xenopus* *tropicalis* REX1-9 **ACTCTCTCTGCAGCTGGATTCTCAACTTCCTGTCCGACAGACGCCAGATGGTTAGACTGGGCAACATCAC** 140

*Gallus gallus* CR1 **CGTGCTGGCTGTCTCGGATCACACGCTCATTCCTCATGTGATCG-AGCATGTCA---TCCAGGAGGATCT** 136

**150 160 170 180 190 200 210**

**....|....|....|....|....|....|....|....|....|....|....|....|....|....|**

*P. ephippifer* Pep-Rex1C1 **GTCGGACACTGTGATTTGTAGTACAGGGGCACCTCAGGGTACAGTCCTGGCTCTCTTCCTGTTCACATTG** 210

*P. ephippifer* Pep-Rex1C19 **GTCGGACACTGTTATTTGTAGTACAGGGGCACCTCAGGGTACAGTCCTGGCTCCCTTCCTGTTCACATTG** 210

*P.* aff. *cuvieri* Alenquer **GTCAGACACTGTGATTTGTAGTACAGGTGCACCTCAGGGTACAGTCCTGGCTCCCTTCCTGTTCACATTG** 208

*P. albifrons* Plb-Rex1 **GTCGGACACTGTGATTTGTAGTACAGGGGCACCTCAGGGTACAGTCCTGGCTCCCTTCCTGTTCACATTG** 210

*P. henselli* Phe-Rex1 **GTCGGACACTGTGATTTGTAGTACAGGGGCACCTCAGGGTACAGTCCTGGCTCCCTTCCTCTTCACATTG** 210

*P. spiniger* Psp-Rex1 **GTCGGACACTGTGATTTGTAGTACAGGTGCACCTCAGGGTACAGTCCTGGCTCCCTTCCTCTTCACATTG** 210

*P. albonotatus* Pab-Rex1C9 **GTTGGACACTGTGATTTGTAGTACAGGTGCACCTCAGAGTACAATCCTGGCTCCCTTTCTCTTCACATTG** 210

*P. albonotatus* Pab-Rex1C6 **GTCGGACACTGTGATTTGTAGTACAGGTGCGCCTCAGGGTACAGTCCTGGCTCCCTTCCTCTTTACATTG** 210

*P. albonotatus* Pab-Rex1C12 **GTTGGACACTG--ATTTATAGTACAGGGGCATCTCAGGGTACAGTCCTG---------------------** 187

*P. ephippifer* Pep-Rex1C11P3**GTCGGACACTGTGATTTGTAGTACAGGTGCGCCTCAGGGTACAGTCCTGGCTCCCTTCCTCTTTACATTG** 210

*P. ephippifer* Pep-Rex1C12P3**GTCGGACACTGTGATTTGTAGTACAGGTGCGCCTCAGGGTACAGTCCTGGCTCCCTTCCTCTTTACATTG** 210

*P. ephippifer* Pep-Rex1C13P3**GTCGGACACTGTGATTTGTAGAACAGGTGCACCTCAGGGTACAGTCTTGGCTCCCTTCCTCCTCACATAA** 210

AJ288466.1 clone Rex1-Anj **GTCCGACATGGTTGTCTGCAGCACGGGGGCCCGGCAGGGAACAGTTCTGGCTCCCTTCCTCTTCACCCTC** 189

U18939.1 *B. baikalensis* **GTCAGATCGGGTGTCCTGCAGCACGGGGGCTCCACAGGGAACCGTTCTGGCTCCATTCCTGTTCTCCATT** 210

AJ288450.1 clone Rex1-Xih **GTCTGATGTGGTGGTCAGTGACGTAGGAGCTCCACAGGGAACTGTGCTTTCTCCCTTTCTCTTCACCTTG** 186

AJ288444.1 clone Rex1-Xih **GTCCGATGTGGTGGTCAGTGACGTAGGAGCTCCACAGGGAACTGTGCTTTGTCCCTTTCTCTTCACCCTG** 189

AJ288442.1 clone Rex1-Xih **GTCTGATGTGGTGGTCAGTGACGTAGGAGCTCCACAGGGAACTGTGCTTTCTCCCTTTCTCTTCACCCTG** 189

*Poeciliopsis gracilis* Rex1 **ATCAGACCATCTAAAATGTAACATTGGAGCTCCTCAAGGAACTGTGCTGTC------GCTATTCACCACC** 183

*Xenopus* *tropicalis* REX1-5 **GTCTGAGACCCTGATCTGTAGTACGGGAGTTCCCCAGGGGACCGTACTGTCTCCGTTTCTTTTTACACTT** 210

*Xenopus* *tropicalis* REX1-2 **CTCATCCACACTGACACTTAGCACCGGTGCCCCCCAGGGATGTGTGCTCAGCCCCCTGCTGTACACCCTG** 210

*Xenopus* *tropicalis* REX1-9 **CTCATCCTCCGTCATAATGAACACTGGTGCTCCACAGGGGTGTGTACTAAGCCCTCTACTGTACTCACTT** 210

*Gallus gallus* CR1 **GTTCCATGATCTTCCCAGGCACAGAGGTGAGACTCACCGGCCTGTAGTTCCCTGGGTCCTCCTTGCTCCC** 206

**220 230 240 250 260 270 280**

**....|....|....|....|....|....|....|....|....|....|....|....|....|....|**

*P. ephippifer* Pep-Rex1C1 **TACACAGCAGACTTTAG--GTACAACTCGACTAGCTGCTACCTCCA-GAAGTTCTCTGATGACTCTGCTA** 277

*P. ephippifer* Pep-Rex1C19 **TACACAGCAGACTTTAG--GTACAATTCAACTAGCTGCTACCTCCA-GAAGTTCTCTGATGACTCTGCTA** 277

*P.* aff. *cuvieri* Alenquer **TACACAGCAGACTTTAG--GTACAATTCATCTAGCTGCTACCTCCA-GAAGTTCTCTGATGACTCTGCTA** 275

*P. albifrons* Plb-Rex1 **TACACAGCAGACTTTAG--GTACAACTCAACTAGCTGCTACCTGCA-GAAGTTCTCTGATGACTCTGCTA** 277

*P. henselli* Phe-Rex1 **TACACAGCAGACTTTAG--GTACAATTCAACTAGCTGCTACCTTCA-GAAGTTCTCTGATGACTCTGCCA** 277

*P. spiniger* Psp-Rex1 **TACACAGCAGACTTTAG--GTACAATTCAACTAGCTGCTACCTCCA-GAAGTTCTCTGATGACTCTGCTA** 277

*P. albonotatus* Pab-Rex1C9 **TACACAGCAGACTTTAG--GTACAATTCAACTAGCTGCTACATCCA-GAAGTTCTCGGATGGCTCTGCTA** 277

*P. albonotatus* Pab-Rex1C6 **TACACGGCGGACTTTAG--GTACAATTCACCTAGCTGCTACCTTCA-GAAGTTCTCTGATG------CTA** 271

*P. albonotatus* Pab-Rex1C12 **----------------------------------------------------------------------** 187

*P. ephippifer* Pep-Rex1C11P3**TACACGGCGGACTTTAG--GTACAATTCACCTAGCTGCTACCTTCA-GAAGTTCTCTGATG------CTA** 271

*P. ephippifer* Pep-Rex1C12P3**TACACGGCGGACTTTAG--GTACAATTCACCTAGCTGCTACCTTCA-GAAGTTCTCTGATG------CTA** 271

*P. ephippifer* Pep-Rex1C13P3**TACACAGCAGACTTTAG--GTACAATTCAACTAGCTGCTACCTTCA-GAACTTCTGTGATGACTCTGCTA** 277

AJ288466.1 clone Rex1-Anj **TGCACTGCAGACTTCAC--GCACAACTCAGCTAACTGCCACCTGCA-GAAGTTCTCTGATGACTCCGCAA** 256

U18939.1 *B. baikalensis* **TACACCTCGGACTTTAA--ACACAACTCTGCCAACTGCCACCTGCA-AAAGTTCTCTGACGACTCTGCAA** 277

AJ288450.1 clone Rex1-Xih **TACACCACTGATTTCCA--GTACAACTCTGAGTCATGTCACCTACA-GAAGTTTTCTGATGACTCAGCGG** 253

AJ288444.1 clone Rex1-Xih **TACACCACTGATTTCCA--GTACAACTCTGAGTCATGTCACCTACA-GAGGTTTTCTGATGACTCAGCGG** 256

AJ288442.1 clone Rex1-Xih **TACACCACTGATTTCCA--GTACAACTCTGAGTCATGTCACCTACA-GAAGTTTTCTGATGACTCAGCGG** 256

*Poeciliopsis gracilis* Rex1 **TACACTGCAGACTTTAA--GTACTGTTCCAAACTGTACCATCTGCA-AAAGTACTCGGATGACATGGCTA** 250

*Xenopus* *tropicalis* REX1-5 **TATACATCCGACTTTAG--ATATAACTCGGAGTCATGCCATCTGCA-GAAGTTTTCTGATGACTCTGTGG** 277

*Xenopus* *tropicalis* REX1-2 **TTCACCCACGACTGTAC--A-GCAACACACAGCTCCAATACTATCATCAAATTTGCAGACGACACCACCA** 277

*Xenopus tropicalis* REX1-9 **TACACATATGACTGCAC--G-GCCACTAGCAGCTCCAACATCGTTGTGAAGTTTGCGGACGACACAACAG** 277

*Gallus gallus* CR1 **TTTCTTGTAAATGGGAGTGACGTAACCCTTCCTCCAGTCATCTGGG--ACCTCGCCTGACAGCCATGACT** 274

**290 300 310 320 330 340 350**

**....|....|....|....|....|....|....|....|....|....|....|....|....|....|**

*P. ephippifer* Pep-Rex1C1 **TAGTTGGCCTCATTACCGGGGACAACGACAGAGAGTACAGAGAACTGACCCGGGGATTTGTGGACTGGTG** 347

*P. ephippifer* Pep-Rex1C19 **TAGTCGGCCTCATTAC-GGGGACAACGACAGAGAGGACAAAGAACTGACCCGGGGATTTGTGGATTGGTG** 346

*P.* aff. *cuvieri* Alenquer **TAGTCGGCCTCATTACCGGGGACAATGACAGAGAGTACAAAGAACTGACCCGGGGATTTGTGGATTGGTG** 345

*P. albifrons* Plb-Rex1 **TAGTCGGCCTCATTACCGGGGACAACGACAGAGAGTACAGAGAACTGACCCGGGGATTTGTGGACTGGTG** 347

*P. henselli* Phe-Rex1 **TAGTAGGCCTCATTACCGGGGACAACGACAGGGAGTACAGAGAATTGACCCGGGGATTTGTGGACTGGTG** 347

*P. spiniger* Psp-Rex1 **TAGTAGGCCTCATTACCGGTGACAACGACAGGGAGTACAGAGAAGTGACCCGGGGATTTGTGGATTGGTG** 347

*P. albonotatus* Pab-Rex1C9 **TAGTCGGCCTCATTACCGGAGAGAACGACAGGGAGTTCAAAGAATTGACCCAAGGATTTGTGGACTGGTG** 347

*P. albonotatus* Pab-Rex1C6 **TAGTCGGCCTCATTACCGACGAGAACAACAGGGAGTACAGAGAACTGACCTGGGGATTTGTGGATTGGTG** 341

*P. albonotatus* Pab-Rex1C12 **----------------------------------------------------------------------** 187

*P. ephippifer* Pep-Rex1C11P3**TAGTCGGCCTCATTACCGACGAGAACAACAGGGAGTACAGAGAACTGACCTGGGGATTTGTGGATTGGTG** 341

*P. ephippifer* Pep-Rex1C12P3**TAGTCGGCCTCATTACCGACGAGAACAACAGGGAGTACAGAGAACTGACCTGGGGATTTGTGGATTGGTG** 341

*P. ephippifer* Pep-Rex1C13P3**TTGTAGGCCTCATTACCAGCGACAACGACAGGGAGTACAGAGAATTGACCCGGGGATTTGTGGATTGGTG** 347

AJ288466.1 clone Rex1-Anj **TCGTCAGCTTYATCACAGATGGGGACGACAGGGAGTACAGAGATCTGACCCAGGGCTTTGTGGATTGGTG** 326

U18939.1 *B. baikalensis* **TCGTCGGCCTCATCTCTGCCGATGATGACAGGGAGTACAGGGAACTTAATCAGGACTTTTTAGGATGGTG** 347

AJ288450.1 clone Rex1-Xih **TTGTCGGGTGTATAGGGGATGGAGGGGAGGGGGAGTACAGGACACTGGTGGACAGCTTTGTGGAGTGGTC** 323

AJ288444.1 clone Rex1-Xih **TTGTCGGGTGTATAGGAGATGGAGGGGAGGGGGAGTACAGGACACTGGTGGACAGCTTTGTGGAGTGGTC** 326

AJ288442.1 clone Rex1-Xih **TTGTCGGGTGTATAGGGGATGGAGGGGAGGGGGAGTACAGGACACTGGTGGACAGCTTTGTGGAGTGGTC** 326

*Poeciliopsis gracilis* Rex1 **TCATGGGGTGTGTGGAAGGGGGAGAGGAGGTGGAGTACAAGACATTGGTGGATAATTTTGTGACATGATG** 320

*Xenopus* *tropicalis* REX1-5 **TTGTTGGGTGTATAAAGGAGGGTGATACCTCTGAGTACCAGTCTGTTGTGGATGACTTTGTTTCTTGGTG** 347

*Xenopus* *tropicalis* REX1-2 **TCATCGGCTGCATTTCTGATGGAGATGAGTCGGCTTACAGGGCAGAGGTGAGAGCCCTGACATCATGGTG** 347

*Xenopus* *tropicalis* REX1-9 **TGGTGGGTCTCATCACCAACGGTGATGAGACGGCATACAGGGAGGAGGTCAATGCCCTGACACATTGGTG** 347

*Gallus gallus* CR1 **TCTCAAATATGATGGAGAGTGGCTCAGCAACCACCT-CAGCCAGCTCCTTCAGAACCCTG-GGATGCACA** 342

**360 370 380 390 400 410 420**

**....|....|....|....|....|....|....|....|....|....|....|....|....|....|**

*P. ephippifer* Pep-Rex1C1 **CCAGCAAAACCATCTTAGGATAAATGGTGGGAAGACCAAGGAGATGGTGGTGGACTTCCGGAAG------** 411

*P. ephippifer* Pep-Rex1C19 **CCAGCAAAACCATCTTAAGATAAATGCGGGGAAGACCAAGGAGATGGTGGTGGACTTCCGGAAG------** 410

*P.* aff. *cuvieri* Alenquer_ **CCAGCAAAACCATCTTAATATAAATGCGGGGAAGACCAAGGAGATGGTGGTGGACTTCCGGAAG------** 409

*P. albifrons* Plb-Rex1 **CCAGCAAAACCATCTTGAGATAAATGCAGGGAAGACCAAGGAGATGGTGGTGGACTTCTGTAAG------** 411

*P. henselli* Phe-Rex1 **CCAGCAAAACCACCTTAGGATAAATGCTGGGAAGACCAAGGAGATGGTGGTGGACTTCCGTAAG------** 411

*P. spiniger* Psp-Rex1 **CCAGCAAAACCACCTCAGGATAAATGCTGGGAAGACCAAGGAGATGGTGGTGGACTTCCGTAAG------** 411

*P. albonotatus* Pab-Rex1C9 **CCAGCAAAACCATCCTAGGATAAATGGTGGGAAGATCAAGAAGATGGTGGTTGACTTCCGAAAG------** 411

*P. albonotatus* Pab-Rex1C6 **CCAGCAAAACCATCTTAGGATAAATGCTGGGAAGACCAAGGAGATGGCGGTGGACTTCCGTAAG------** 405

*P. albonotatus* Pab-Rex1C12 **---------------------------------------------------------------G------** 188

*P. ephippifer* Pep-Rex1C11P3**CCAGCAAAACCATCTTAGGATAAATGCTGGGAAGACCAAGGAGATGGCGGTGGACTTCCGTAAG------** 405

*P. ephippifer* Pep-Rex1C12P3**CCAGCAAAACCATCTTAGGATAAATGCTGGGAAGACCAAGGAGATGGCGGTGGACTTCCGTAAG------** 405

*P. ephippifer* Pep-Rex1C13P3**CCAGCAA-----------------TGCTGGGAAGACCAAGGAGATGGTGGTGGACTTCCGTAAG------** 394

AJ288466.1 clone Rex1-Anj **CCAGTGGAACTGCCTCCAGCTCAATGCAGGGAAAACCAAGGAGCTGGTGGTGGACTTCCGAAGG------** 390

U18939.1 *B. baikalensis* **CCAGCGGAACCGCCTCCAGATAAACTCCAGTAAGACCAAGGAGCTGGTGGTGGACTTCCGCCGG------** 411

AJ288450.1 clone Rex1-Xih **TGAGCAGAATCACCTGAGGCTCAACATTAGTAAGACCAGAGAGATGGTGATTGACTTCAGAAGG------** 387

AJ288444.1 clone Rex1-Xih **TGACCAGAATCACCTGAGGCTCAACATTAGTAAGACCAGAGAGATGGTGATTGACTTCAGAAGG------** 390

AJ288442.1 clone Rex1-Xih **TGAGCAGAATCACCTGAGGCTCAACATTTGTAAGACCAGAGAGATGGTGATTGACTTCAGAAGG------** 390 *Poeciliopsis gracilis* Rex1 **AAAAGAAAACCAACTTCTGCTTAATGTAAACAAGACCAAGGAAATGGTTGTCAGTTTCTGGAAC------** 384

*Xenopus* *tropicalis* REX1-5 **TGAGTGTAATTACTTACAACTGAACATCAGTAAAACCAGGGAGATGGTTGTAGATTTTGGGAGA------** 411

*Xenopus* *tropicalis* REX1-5 **CCGGGACAACAACCTGCTGCTCAACGTCAGCAAAACTAAGGAGCTCATTGTGGATTACAGGAGAC-----** 412

*Xenopus* *tropicalis* REX1-9 **CCAGGAAAATCATCTCTCCCTCAATGTCGGAAAAACCAAAGAGCTGATAGTGGACTTCCGGAGG------** 411

*Gallus gallus* CR1 **CCATCCGGCCC--TGAAGACTTGTACTCATCCAGTCTCATGAGGCGGTCTCGGACTTGCTCTGCCCTTAG** 410

**430 440 450 460 470 480 490**

**....|....|....|....|....|....|....|....|....|....|....|....|....|....|**

*P. ephippifer* Pep-Rex1C1 **CGCA---GTCCT--TTCTCATCCAGTGGTCATCCA--GGGAACAGAGATTGAGGTAGTGAGGTCTT-ATA** 473

*P. ephippifer* Pep-Rex1C19 **TTCA---GTCCTCCTTCTCATCCAGTGGTCATCCA--GGGAACAGACATTGAGGTAGTGAGGTCTT-ATA** 474

*P.* aff. *cuvieri* Alenquer_ **CGCA---GTCCTCCTTCTCATCCAGTGGTCATCCA--GGGGTCGAACATTGAGGTAGTGAAGACCT-ACA** 473

*P. albifrons* Plb-Rex1 **CACA---GTCCTCCTTCTCATCCAGTGGTCATCCA--GGGGGCGGACATTGAGGTAGTGAAGACCT-ACA** 475

*P. henselli* Phe-Rex1 **CGCA---GTTCTCCTTCTCAACCAGTGGTCATCCA--GGGGGCGGACATTGAGGTAGTGAAGTCCT-ATA** 475

*P. spiniger* Psp-Rex1 **CGCA---GTCCTCCTGCTCATCCAGTGGTCATCCA--GGGGACGGACATTGAGGTAGTGAAGTCCT-ATA** 475

*P. albonotatus* Pab-Rex1C9 **CACA---GCCCTCCTTCTCAACCAGTTGTCATCAA--GAGGATGGACATTGAGGTAGTGAAGTCCT-ATA** 475

*P. albonotatus* Pab-Rex1C6 **CGCA---GT-----------------GGTCATCCA--GGGGACGGACATTGAGGTAGTGAAGTCCT-ATA** 452

*P. albonotatus* Pab-Rex1C12 **CGGA---GTCCTCCTTCTCAACCAGTGGTCATCCA--GGGGACGGACATTGAGGTAGAGAAGT-------** 246

*P. ephippifer* Pep-Rex1C11P3**CGCA---GT-----------------GGTCATCCA--GGGGACGGACATTGAGGTAGTGAAGTCCT-ATA** 452

*P. ephippifer* Pep-Rex1C12P3**CGCA---GT-----------------GGTCATCCA--GGGGACGGACATTGAGGTAGTGAAGTCCT-ATA** 452

*P. ephippifer* Pep-Rex1C13P3**CGCT---TGCCTCCTTCTAAACCAGTGGTCATCCA--GGGGACGTACATTGAGGTAGTGAAGTTCT-ATA** 458

AJ288466.1 clone Rex1-Anj **TGCCAACAATCGCCTCCTATACCAGTGAACATCCA--GGGAATGGAAATTGAGATGGTGAAATCTT-ACA** 457

U18939.1 *B. baikalensis* **GGCAAACGCTCTCCTCCGCTACCATTGAGCATCCA--GGGACTGGACATTGAGATGGTGACATCTT-ACA** 478

AJ288450.1 clone Rex1-Xih **AAGA---AGATACCTTCACGGCCACTGAAGATCAAA--GGGGAAGTGGTGGAGGAGGTGGAGGATT-ACA** 451

AJ288444.1 clone Rex1-Xih **AAGA---AGACATCTTCACGGCCACTGAAGATCAAA--GGGGAAGTGGTGGAGGAGGTGGAGGATT-ACA** 454

AJ288442.1 clone Rex1-Xih **AAGA---AGACATCTTCACGGCCACCGAAGATCAAA--GGGGAAGTGGTGGAGGAGGTGGAGGACT-ACA** 454

*Poeciliopsis gracilis* Rex1 **AAAA---AATNACATTTGCTCCCAATCTACATCTCAC--GGACAGATGTGGAGCAGGTTCAGTCCT-ACA** 448

*Xenopus* *tropicalis* REX1-5 **AGCA---AGATTCATGTGACTCCTATATCTGTTAA--GGGGGAAATAGTGGATATGGTTTCTGATT-ATA** 475

*Xenopus* *tropicalis* REX1-2 **TGCAGGGAGGAGGCCATACCCCCA-TTCACATTGA--GGGAGCAGAGGTGGAGAGAGTCAGCTGCT-TCA** 478

*Xenopus* *tropicalis* REX1-9 **TGCA--GAGGTGCACATTCCCCCA-TCATCATCAAC--GGTGCTGCTGTGGAGAGAGTGAACAGCT-TCC** 475

*Gallus gallus* CR1 **TGTGGGGGGGGATTTACCCCCCTGGTCCCCACCTG---GAGGCTTGGGGGTGCAGGGCTCAGGGACGTGA** 477

**500 510 520 530 540 550 560**

**....|....|....|....|....|....|....|....|....|....|....|....|....|....|**

*P. ephippifer* Pep-Rex1C1 **AGTACTTGGGTGTCCTCCTAAACAATAAACTGGATTGGGCTGAGAACATAAATGCGCTTCAGAGGAAGGG** 543

*P. ephippifer* Pep-Rex1C19 **AGTACTTGGGTGTCCTCCTAAACAATAAACTGGATTGGGCAGAGAACATAAATGCGCTTCAGAGGAAGGG** 544

*P.* aff. *cuvieri* Alenquer_ **AGTACTGGGGGGTCCTCCTAAACAATAAACTGGATTGGGCCGAGAACATAAATGCGCTTCAAAGGAAGGG** 543

*P. albifrons* Plb-Rex1 **AGTACTTGGGTGTCCTCCTAAACAATAAACTGGATTGGGCAGAGAACATAAATGCGCTTCAGAGGAAGGG** 545

*P. henselli* Phe-Rex1 **AGTACTTGGGTGTCCTCCTCAACAATAAACTGGATTGGGCAGAGAACATAAATGCGCTTCAGAGGAAGGG** 545

*P. spiniger* Psp-Rex1 **AGTACTTGGGTGTCCTCCTCAACAATAAACTGGATTGGGCAGAGAACATAAATGCGCTTCACAGGAAGGG** 545

*P. albonotatus* Pab-Rex1C9 **AGTACTTGGGTGTCCTCCTCAACAATAAACTGGATTGGGCAGAGAACATAAATGCACTTCAGAGGAAGGG** 545

*P. albonotatus* Pab-Rex1C6 **AGTACTC-GGTGTCCTCCTCAACAATAAANGGGATTGGGCTAAGAACATAAATGCGCTTCAGAGGAAGGG** 521

*P. albonotatus* Pab-Rex1C12 **---ACTTCTGTGTCCTCCTAAACAGTAAACTGGATTGGGCAGAGAACATAAATGCACTTCACAGGAAGGG** 313

*P. ephippifer* Pep-Rex1C11P3**AGTACTC-GGTGTCCTCCTCAACAATAAACAGGATTGGGCAGAGAACATAAATGCACTTCACAGGAGGGG** 521

*P. ephippifer* Pep-Rex1C12P3**AGTACTC-GGTGTCCTCCTCAACAATAAACAGGATTGGGCAGAGAACATAAATGCACTTCACAGGAAGGG** 521

*P. ephippifer* Pep-Rex1C13P3**AGTACTTGGGTGTCCTCCTCAACAATAAACTGGATTGGACGGAGAACATAAACGCACTTCACAGGAAGGG** 528

AJ288466.1 clone Rex1-Anj **AGTACCTGGGTGTTCACCTGAATAATAAACTGGACTGGACTGACGATACAAATGCACTATATAAGAAAGG** 527

U18939.1 *B. baikalensis* **AGTACCTGGGTGTTCACTTGAACAATAAACTGGACTGGTCCGACCACGCGCATGCGCTTTATAAAAAGGG** 548

AJ288450.1 clone Rex1-Xih **AATACCTGGGAGTTGTAATCGGCAACAGACTGGACTGGGCATCTAACACTGACGCTGTGTGCAAGAAGGG** 521

AJ288444.1 clone Rex1-Xih **AATACCTGGGAGTTGTAATCGACAACAGACTGGACTGGGCATCTAACACTGACGCTGTGTGCAAGAAGGG** 524

AJ288442.1 clone Rex1-Xih **AATACCTGGGAGTTGTAATCGGCAACAGACTGGACTGGGCGTCTAACACTGACGCTGTGTGCAAGAAGGG** 524

*Poeciliopsis gracilis* Rex1 **AGTACCTTGGTGTTACATTAAACAGCAAGCTACAGTGGTCTGCAAACTCAAATGTGCGCTACAAAAAGGC** 518

*Xenopus* *tropicalis* REX1-5 **AATATCTGGGAGTCCACTTGGACAGTAAACTTGATTGGTCACTTAATACAATGGCACTTTATAAAAAAGG** 545

*Xenopus* *tropicalis* REX1-2 **GATTCCTGGGCATCAATATCAGTGAGGATCTGAGTTGGTCTCACCATGTTGGTGTGATCACAAAAGCTGC** 548

*Xenopus* *tropicalis* REX1-9 **GTTTCCTGGGAGTTCACATCGCAGATGATCTCACATGGTCAGTTCATATTGACAAAACAGTGAAGAAGGC** 545

*Gallus gallus* CR1 **GAAAGACTGGAATCCTGGCCTCCAGTGAAGACTGAGGCAAAGAACTCATTCAG-----------------** 530

**570 580**

**....|....|....|....|....|.**

*P. ephippifer* Pep-Rex1C1 **TCAGAGCAGACTCTTTCTGCTGAGGG** 569

*P. ephippifer* Pep-Rex1C19 **TCAGAGCAGACTCTTTCTGCTGAGGG** 570

*P.* aff. *cuvieri* Alenquer_ **TCAGAGCTACTCTTTTCTGCTGAGGG** 569

*P. albifrons* Plb-Rex1 **TCAGAGCAGACTCTTTCTGCTGAGGG** 571

*P. albifrons* Plb-Rex1 **TCAGAGCAGACTCTTTCTGCTGAGGG** 571

*P. spiniger* Psp-Rex1 **TCAGAGCAGACTCTTTCTGCTGAGGG** 571

*P. albonotatus* Pab-Rex1C9 **TCAGAGCAGACTCTTTCTGCTGAGGG** 571

*P. albonotatus* Pab-Rex1C6 **TCAGAGCAGACTCTTTCTGCTGAGGG** 547

*P. albonotatus* Pab-Rex1C12 **TCAGAGCAGACTCTTTCTGCTGAGGG** 339

*P. ephippifer* Pep-Rex1C11P3**TCAGAGCAGACTCTTTCTGCTGAGGG** 547

*P. ephippifer* Pep-Rex1C12P3**TCAGAGCAGACTCTTTCTGCTGAGGG** 547

*P. ephippifer* Pep-Rex1C13P3**TCAGAGCAGACTCTTTCTGCTGAGGG** 554

AJ288466.1 clone Rex1-Anj **AC------------------------** 529

U18939.1 *B. baikalensis* **ACAGAGCAGACTCTTTCTGCTGAGGA** 574

AJ288450.1 clone Rex1-Xih **AT------------------------** 523

AJ288444.1 clone Rex1-Xih **AT------------------------** 526

AJ288442.1 clone Rex1-Xih **AT------------------------** 526

*Poeciliopsis* gracilis Rex1 **CA------------------------** 520

*Xenopus tropicalis* REX1-5 **ACAGAGTCGGTTGTATTTTTTGCGGA** 571

*Xenopus tropicalis* REX1-2 **AAGACAGCGGCTCTTCTTTC------** 568

*Xenopus tropicalis* REX1-9 **GCAGCAGCGACTCTTCTTTCTCAGGA** 571

*Gallus gallus* CR1 **----TACCTCCGCTTTCTCTTCATCC** 552
